# Association Between Advanced Glycation End-Products and Adherence to the Mediterranean Diet in Individuals with Type 2 Diabetes Mellitus: A Cross-Sectional Study

**DOI:** 10.3390/nu18121887

**Published:** 2026-06-11

**Authors:** Maria Patsiliva, Aikaterini Theodorakopoulou, Anastasia Stergioti, Eleni Rebelos, Evangelos Liberopoulos, Ioanna A. Anastasiou, Nikolaos Tentolouris

**Affiliations:** 1 Diabetes Center, First Department of Propaedeutic Internal Medicine, Laiko General Hospital, Medical School, National and Kapodistrian University of Athens, 11527 Athens, Greece; mpatsiliva2@gmail.com (M.P.); kate.theodorakopoulou@gmail.com (A.T.); st.anastasia@yahoo.gr (A.S.); eleni.rebelos@utu.fi (E.R.); vaglimp@yahoo.com (E.L.); 2 Department of Clinical and Experimental Medicine, University of Pisa, 56126 Pisa, Italy; 3 Department of Pharmacology, Medical School, National and Kapodistrian University of Athens, 11527 Athens, Greece

**Keywords:** diabetes mellitus, advanced glycation end-products, Mediterranean diet, Mediterranean diet adherence, MedDietScore, glycemic control, autofluorescence

## Abstract

**Background/Objectives**: Advanced glycation end-products (AGEs) are implicated in the vascular complications of type 2 diabetes mellitus (T2DM). Although the Mediterranean diet (MD) confers well-recognized benefits, adherence among people with T2DM is moderate. Evidence regarding the relationship between adherence to the MD and AGEs remains limited. This study aimed to investigate the association between adherence to the MD and AGE levels in individuals with T2DM in Greece. **Methods**: Adults with T2DM were enrolled in this cross-sectional study. Ocular and skin AGEs were assessed using autofluorescence techniques, while serum AGEs were quantified using an enzyme-linked immunosorbent assay (ELISA). MD adherence was scored with the Mediterranean Diet Score (MedDietScore) (range 0–55). **Results**: Sixty-one individuals were studied (mean age: 65 ± 9 years; 35 men). Median glycated hemoglobin (HbA1c) and MedDietScore were 6.95 (6.2–7.5)% and 29.0 (27.5–31.5), respectively. Higher adherence to the MD was more frequent among women (*p* = 0.019) and was associated with lower HbA1c levels (*p* = 0.002). MedDietScore was inversely correlated with HbA1c (*ρ* = −0.437, *p* < 0.001), ocular AGEs (*ρ* = −0.435, *p* = 0.013), and skin AGEs (*ρ* = −0.309, *p* = 0.033). In multivariable regression analyses, higher adherence to the MD was independently associated with lower ocular (*p* = 0.043) and skin AGEs (*p* = 0.005). **Conclusions**: Higher adherence to the MD was associated with female sex and lower HbA1c. MedDietScore was inversely related to skin, and ocular AGE levels in individuals with T2DM.

## 1. Introduction

Diabetes mellitus (DM) is a chronic metabolic condition in which circulating glucose remains elevated owing to inadequate insulin secretion, diminished insulin action, or both [1,2]. DM represents a major global health issue, as according to the International Diabetes Federation [IDF] Atlas, in 2024, approximately 589 million people, or 1 in 9 individuals aged 20–79 years, were living with the disease [3]. Type 2 diabetes mellitus (T2DM), which accounts for 90–95% of cases worldwide, is the predominant form [1]. In Greece, the burden of diabetes is also considerable; based on IDF estimates, the prevalence of diabetes among Greek adults is 11.4% [3]. Notably, data from the Hellenic National Nutrition and Health Survey indicate a prevalence of 13.7% in adults aged over 60 years [4]. Although the pathophysiology of T2DM is complex, involving genetic, biological, behavioral, and social factors, the primary mechanisms include β-cell dysfunction, leading to progressive insulin deficiency, and insulin resistance [5,6]. T2DM causes vascular damage, resulting in microvascular and macrovascular complications that are associated with impaired quality of life and adverse clinical outcomes [7].

Although advanced glycation end-products (AGEs) form gradually as part of normal aging [8], chronic hyperglycemia significantly accelerates their generation, contributing to the development of diabetic complications [9]. Chemically, they comprise a diverse family of molecules produced via the Maillard reaction—a non-enzymatic, multi-step process in which reducing sugars react with the free amino groups of macromolecules, ultimately yielding the irreversible compounds collectively termed AGEs [10,11].

AGEs exert their effects through cross-linking with extracellular matrix proteins, altering tissue structure and function [10,12]. In addition, AGEs engage the receptor for AGEs (RAGE), triggering pro-inflammatory pathways and a rise in reactive oxygen species that drives oxidative stress [13,14].

Apart from endogenous formation, AGEs enter the body from exogenous sources, including tobacco smoke and diet. The Western dietary pattern and food processing methods constitute important sources of dietary AGEs [9,13,14]. Conversely, greater adherence to the Mediterranean diet (MD) has been linked to lower AGE concentrations [15]. The MD is also considered one of the most appropriate dietary patterns for diabetes management [16,17]. Its profile emphasizes plant-derived foods, including unrefined cereals, fruits, vegetables, legumes, nuts, seeds, and extra virgin olive oil; moderate consumption of fish, low-fat dairy products, poultry, eggs, and wine; and a low consumption of red meat, processed foods, and added sugars [17,18,19,20,21]. Beyond this composition, MD adherence has been associated with lower oxidative stress and low-grade inflammation, improved insulin sensitivity, and better glycemic control. In addition, adherence to the MD has been linked to improved cardiovascular health, including a more favorable lipid profile, better blood pressure regulation, and improved indices of central adiposity [17,20,21,22,23,24,25].

Even though the MD’s benefits are well-established, adherence among individuals with T2DM is generally moderate, as reported in the systematic review by Ayoub et al. [26]. In the present study, adherence was classified as low, moderate, or high based on the MedDietScore tertiles (see Section 3.2). Moreover, evidence on the interplay between MD adherence and AGEs remains limited. Previous studies have shown an inverse relationship between adherence and skin AGEs both in middle-aged adults without known cardiovascular disease or T2DM (*p* = 0.026) [27], as well as in healthy populations (*p* < 0.05) [28]. In addition, following the MD pattern has also been tied to lower circulating AGE levels in the postprandial state [29]. In clinical populations, MD-related declines in circulating AGEs have accompanied T2DM remission among newly diagnosed patients with coronary heart disease (CHD) [30] and the preservation of kidney function in individuals with T2DM and CHD [31]. Furthermore, in individuals with T2DM in Croatia, AGE levels measured via skin autofluorescence (SAF) were inversely associated with MD adherence [32].

However, evidence regarding the simultaneous evaluation of tissue-bound and circulating AGEs and their association with MD adherence in individuals with T2DM remains scarce. Accordingly, the present study set out to examine how MD relates to skin, ocular, and serum AGEs among adults with T2DM in Athens, Greece. In addition, demographic, anthropometric, clinical, and biochemical characteristics were recorded.

## 2. Materials and Methods

### 2.1. Study Design and Participants

The study followed a cross-sectional design and took place at Laiko General Hospital, Athens, Greece, between April and December 2025. It adhered throughout to the principles of the Declaration of Helsinki [33]. The protocol received approval by the Research Ethics Committee of the National and Kapodistrian University of Athens (Approval Number: 5082/10-04-2025). Each participant gave written informed consent after being fully briefed on the study procedures.

Consecutive adults diagnosed with T2DM, according to the diagnostic criteria of the American Diabetes Association [1], who were regular attendees of the outpatient Diabetes Clinic of the First Propaedeutic Department of the hospital, were eligible for the study.

Participants were ineligible if they had a non-T2DM form of diabetes, microvascular complications (nephropathy, neuropathy, or retinopathy) of non-diabetic etiology, advanced liver disease, severe peripheral arterial disease [ankle–brachial index (ABI) < 0.4], and severely reduced estimated glomerular filtration rate (eGFR) (<30 mL/min/1.73 m^2^). Individuals with advanced heart failure (New York Heart Association class III or IV), atrial fibrillation, atrial flutter, pacemaker implantation, or a history of arrhythmia were also excluded. In addition, individuals with a history of ocular trauma, ocular surgery, glaucoma, or contact lens use, as well as those with malignancy, neurologic impairment, rheumatic, hematologic, or autoimmune diseases were excluded. The presence of fever at the time of assessment, a documented history of chronic excessive alcohol consumption (i.e., habitual heavy drinking or a recorded diagnosis of alcohol use disorder, based on the medical history), and current treatment with corticosteroids or immunosuppressants were also considered exclusion criteria.

### 2.2. Study Procedures

All participants presented at the Diabetes Center of the hospital early in the morning. They were instructed to fast for 12 h, abstain from smoking, and take any medication after completion of the visit. The visit included an interview to collect information on demographic (e.g., sex, age) and behavioral characteristics (e.g., smoking habits). Diabetes-related parameters were obtained from the participants’ medical records by the study investigators, including disease duration, pharmacotherapy, and presence of microvascular or macrovascular complications. Information on comorbidities and additional treatment was also retrieved from their medical history.

#### 2.2.1. Anthropometric Measurements

Trained staff carried out all anthropometric assessments. With participants in light clothing and without footwear, body weight was recorded to the nearest 0.1 kg using an electronic scale. Height was measured to the nearest 0.1 cm using a non-portable stadiometer and recorded in meters (m). Body mass index (BMI) was derived as weight (kg) divided by height squared (m^2^). Participants were grouped into normal weight, overweight, and obese categories based on BMI (18.5–24.99 kg/m^2^, 25.0–29.99 kg/m^2^, and ≥30.0 kg/m^2^, respectively). Waist and hip circumferences were obtained with an inelastic tape [34], and the waist-to-hip ratio (WHR) and waist-to-height ratio (WHtR) were subsequently calculated as additional indices of central adiposity. WHR was calculated as waist circumference divided by hip circumference, and the WHtR as waist circumference divided by height [35,36].

#### 2.2.2. Clinical Data

Fasting blood samples were obtained from every participant to measure glycemic, lipid, renal, and nutritional indices, including plasma glucose, glycated hemoglobin (HbA1c), total cholesterol, high-density lipoprotein (HDL) cholesterol, triglycerides, serum creatinine, 25-hydroxy-vitamin D, ferritin, vitamin B12, and folate. Biochemical indices were measured using an electrochemiluminescence immunoassay (ECLIA) on a COBAS analyzer (Cobas 6000, Roche Diagnostics International Ltd., Rotkreuz, Switzerland). Low-density lipoprotein (LDL) cholesterol concentration was derived with the Friedewald equation [37,38], and eGFR with the 2021 Chronic Kidney Disease Epidemiology Collaboration (CKD-EPI) equation [39]. A morning spot urine sample was also collected from each participant and analyzed at the clinical laboratory of the hospital; the albumin-to-creatinine ratio (ACR) was calculated as the ratio of urinary albumin to urinary creatinine.

#### 2.2.3. Quantification of Circulating AGEs (*n* = 55)

To obtain serum, samples were centrifuged at 4000 rpm for 10 min, and stored at −80 °C until analysis. Serum AGE levels were quantified with a sandwich enzyme-linked immunosorbent assay (ELISA) using a commercial ELISA kit (Human AGE ELISA Kit, EH0622, FineTest, Wuhan, China) according to the manufacturer’s instructions. Serum AGE concentrations (ng/mL) were calculated based on the standard curve.

#### 2.2.4. Assessment of Ocular AGEs (*n* = 32)

Ocular AGE accumulation was evaluated via lens autofluorescence (LAF) [40]. Measurements were performed on the left eye using the ClearPath DS-120^®^ lens fluorescence biomicroscope optical system (Freedom Meditech, Inc., San Diego, CA, USA). This is a non-invasive technique that does not require pupil dilation. During scanning of the eye, scattered and fluorescent light is recorded. The result represents the ratio of LAF to scattered light and is expressed in arbitrary units (AU) [40,41,42]. Lens crystallins are susceptible to AGE accumulation [40], and increased AGE accumulation leads to higher LAF values [43]. In individuals with T2DM, LAF tracks with disease duration and diabetes-related complications [41].

#### 2.2.5. Assessment of Skin AGEs (*n* = 48)

Skin AGEs were measured using SAF with the AGE Reader device (DiagnOptics, Groningen, The Netherlands) [44]. SAF is a non-invasive, simple, and rapid method [45], that exploits the autofluorescence of AGEs bound to dermal tissue [46]. It has been suggested that SAF correlates with AGE levels in skin biopsies, which are considered the gold standard method [44,45,46]. The AGE Reader illuminates approximately 4 cm^2^ of skin on the inner forearm [44]. SAF is calculated based on the intensity of emitted and reflected light, expressed in AU, and displayed on the device screen [46]. SAF has been reported to be higher in individuals with diabetes [46], correlates with HbA1c levels [12], and may predict diabetes-related complications [44].

#### 2.2.6. Assessment of Adherence to the Mediterranean Diet

Dietary intake was assessed at enrollment through a face-to-face interview conducted by trained study investigators, in which the participants reported their usual weekly consumption of each food group over the preceding three months. Adherence was quantified with a modified Mediterranean Diet Score (MedDietScore) [47], adapted from the instrument originally devised by Panagiotakos et al. [48] and validated in Greek populations. In contrast to the original score, which evaluates the monthly consumption of the food groups, the modified version evaluates their weekly consumption, which was considered more representative of habitual intake. Portion sizes were defined according to the national dietary guidelines for adults in Greece [49]. Each of the eleven food groups is rated from 0 to 5: fruits, vegetables, legumes, unrefined cereals, potatoes, olive oil, fish, poultry, red meat and meat products, full-fat dairy products, and alcohol. For components consistent with the MD, higher consumption corresponds to higher scores, whereas for the remaining components, reverse scoring was applied. Alcohol intake was scored separately: a daily intake of <300 mL was assigned a score of 5, while intakes of 300, 400, 500, 600 and >700 mL/day were assigned scores of 4, 3, 2, 1, and 0, respectively. An intake of 0 mL/day was also assigned a score of 0. Total scores ranged from 0 to 55, with higher values denoting closer adherence [47].

#### 2.2.7. Statistical Analysis

Analyses were conducted in the Statistical Package for the Social Sciences (SPSS), version 29.0 (IBM Corp., Armonk, NY, USA). Normality of data distribution was assessed using the Shapiro–Wilk test and graphical methods. Normally distributed continuous variables are presented as mean ± standard deviation (SD), whereas the non-normally distributed variables are presented as median (interquartile range). Categorical variables are presented as absolute (*n*) and relative (%) frequencies. Comparisons between two independent groups were performed using Student’s *t*-test or the Mann–Whitney *U* test, as appropriate. Pearson’s chi-square (*χ*^2^) test was used for categorical variables. Spearman’s rank correlation coefficient (*ρ*) was used to assess non-parametric correlations. Multivariable linear regression analyses were performed to investigate the association between adherence to the MD and AGE levels. Three separate models were constructed for ocular, skin, and serum AGEs as dependent variables. Covariates were selected on clinical grounds and according to the sample available for each AGE measure to avoid model overfitting. Multicollinearity among independent variables was assessed using variance inflation factor (VIF). Results are presented as standardized regression coefficients (*β*) and corresponding *p*-values. A *p*-value < 0.05 was considered statistically significant.

## 3. Results

### 3.1. Sample Characteristics

**Figure 1:**
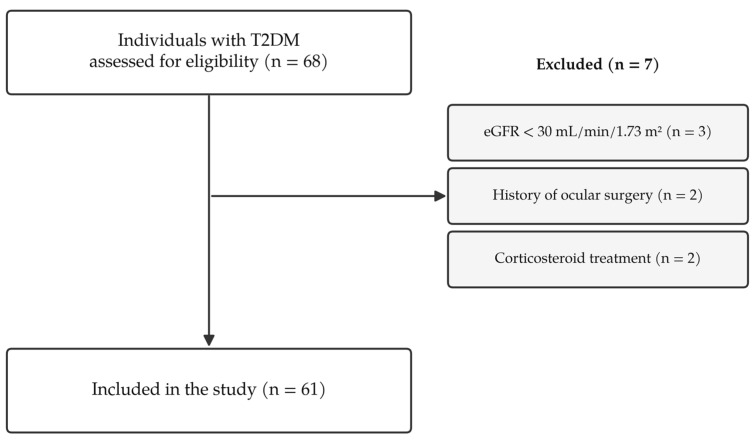
Flow diagram of participant inclusion and exclusion. eGFR: Estimated glomerular filtration rate; T2DM: type 2 diabetes mellitus.

A total of 61 individuals with T2DM were included in this study (Figure 1). Of these, 35 (57.4%) were men, and the mean age was 65 ± 9 years. The median diabetes duration was 14.0 (7.25–23.0) years. With regard to glucose-lowering therapy, 50 participants (82.0%) were receiving metformin, 26 (42.6%) insulin, 34 (55.7%) sodium–glucose cotransporter-2 (SGLT-2) inhibitors, 12 (19.7%) dipeptidyl-peptidase-4 (DPP-4) inhibitors, and 35 (57.4%) glucagon-like peptide-1 receptor agonists (GLP-1 RA). In addition, 73.3% of the study population were non-smokers.

The mean waist and hip circumferences were 106.4 ± 10.6 cm and 103.4 ± 8.04 cm, respectively. The mean WHR was 1.03 ± 0.09, and the mean WHtR was 0.63 ± 0.07. The median BMI was 29.3 (26.5–33.6) kg/m^2^; 41.7% of participants fell within the overweight range and 40.0% within the obesity range.

Regarding laboratory parameters, the median fasting plasma glucose was 120.0 (105.0–147.0) mg/dL and the median HbA1c was 6.95 (6.2–7.5)%. Additional biochemical characteristics are presented in Table 1.

### 3.2. Adherence to the Mediterranean Diet

The median MedDietScore was 29.0 (27.5–31.5). Based on tertiles of the MedDietScore distribution, MD adherence was classified as low, moderate, or high. Accordingly, 20 participants (32.8%) were classified as having low adherence (MedDietScore ≤ 28.0), 26 (42.6%) as having moderate adherence (29 ≤ MedDietScore ≤ 31.0), and 15 (24.6%) as having high adherence (MedDietScore > 31.0). For subsequent comparative analyses, the cohort was dichotomized at the median MedDietScore (≤29.0 vs. >29.0).

### 3.3. Sample Characteristics According to Median MedDietScore

Individuals with higher adherence to the MD were more frequently women (*p* = 0.019), more frequently treated with metformin (*p* = 0.024) and less frequently with insulin (*p* = 0.019), and exhibited higher BMI (*p* = 0.012), waist circumference (*p* = 0.023), and WHtR (*p* = 0.034), as well as lower HbA1c levels (*p* = 0.002). However, the associations observed for adiposity measures were not confirmed in subsequent correlation analyses.

A trend toward lower ocular AGEs was also observed in the higher adherence group; however, this did not reach statistical significance (*p* = 0.071). Skin and serum AGEs did not differ between the two groups. The characteristics of participants according to MD adherence are presented in Table 2.

### 3.4. Associations Between MedDietScore and Demographic, Anthropometric, Clinical, and Biochemical Parameters

Spearman correlation analysis showed significant inverse correlations between MedDietScore and HbA1c (*ρ* = −0.437, *p* < 0.001), ocular AGEs (*ρ* = −0.435, *p* = 0.013), and skin AGEs (*ρ* = −0.309, *p* = 0.033). An inverse correlation was also observed between MD adherence and serum AGEs; however, this did not reach statistical significance (*p* = 0.060).

Detailed results are shown in Table 3.

### 3.5. Multivariable Analysis

Three multivariable linear regression models were constructed with AGEs as the dependent variables and MedDietScore as the main predictor, adjusting for selected covariates.

In the model predicting ocular AGEs (*n* = 32), higher MedDietScore remained independently associated with lower ocular AGE levels after adjusting for age and diabetes duration (*β* = −0.336, *p* = 0.043).

Similarly, in the model predicting skin AGEs (*n* = 48), higher adherence to the MD was independently associated with lower skin AGE levels after adjusting for age, diabetes duration, HbA1c, and smoking status (*β* = −0.387, *p* = 0.005).

In contrast, MedDietScore was not independently associated with serum AGEs (*n* = 55) after adjusting for age, diabetes duration, HbA1c, smoking status, and eGFR (*β* = −0.037, *p* = 0.823). The overall serum AGE regression model was not statistically significant (*p* = 0.352).

In sensitivity analyses additionally adjusting for metformin and insulin use, the inverse association between MedDietScore and skin AGEs remained significant (*β* = −0.346, *p* = 0.013, *n* = 47), and neither metformin (*p* = 0.173) nor insulin (*p* = 0.891) was independently associated with skin AGEs. For ocular AGEs, the standardized coefficient for MedDietScore was essentially unchanged (*β* = −0.338; *n* = 32) but reached only borderline significance (*p* = 0.057), with neither metformin (*p* = 0.391) nor insulin (*p* = 0.257) being an independent predictor.

## 4. Discussion

The present cross-sectional study extends the current literature by simultaneously assessing tissue-bound (skin and ocular) and circulating AGE levels and investigating their associations with adherence to the MD (scored with the MedDietScore) [47,48], in a sample of individuals with T2DM in Athens, Greece. Overall, adherence to the MD in this sample can be characterized as low to moderate. Higher adherence was associated with female sex and lower HbA1c, ocular AGE, and skin AGE levels. In contrast, serum AGEs were not significantly associated with MD adherence. Notably, ocular and skin AGEs remained independently and inversely associated with MD adherence in the multivariable regression models after adjusting for clinically relevant covariates.

The low-to-moderate adherence seen here aligns with reports from Mediterranean T2DM populations [26] and may reflect the gradual transition from traditional to more Westernized dietary patterns [26,50]. Similarly, studies in Greek populations, both in healthy individuals and in individuals with DM, have reported low to moderate adherence to the MD [50,51,52,53].

With regard to demographic characteristics, female sex was associated with higher MD adherence in univariate analyses. However, the available literature reports conflicting results in people with DM [26]. Nevertheless, data from specific cohorts are in agreement with our findings. For instance, in a prospective cohort from the ATTICA study, long-term MD adherence was associated with female sex [54]. Similarly, a study conducted in a healthy Italian population reported higher adherence among women [55]. In contrast, earlier data from Greek populations revealed no sex-related differences in MD adherence [50]. Despite these inconsistencies, both healthy individuals and those with DM appear to exhibit a greater tendency toward healthier dietary patterns among women [55,56].

Our cohort showed no significant link between age and MD adherence, although prior work points to a positive relationship, possibly reflecting a higher likelihood of consuming homemade meals among older individuals [26]. The relatively narrow age span of our participants may account for the null finding.

With regard to clinical parameters, no association was observed between diabetes duration and MD adherence in this study, while the available evidence remains inconclusive [26]. This finding may be explained by the relative homogeneity of the sample, as the median diabetes duration was approximately 14 years, as well as the relatively small sample size.

Among the biochemical parameters, HbA1c was inversely associated with MD adherence in the present sample. Similar findings have been reported in previous research supporting a favorable effect of the MD on glycemic control [24,57,58,59,60], including a recent study in adults with T2DM in Greece [53].

The MD’s favorable effect on glycemic regulation may be attributed to improvements in insulin sensitivity [59], reduced glycemic variability [15], and lower oxidative and inflammatory burden [25,61]. These actions probably arise from the diet’s makeup—rich in dietary fiber, vitamins (e.g., vitamin C), unsaturated fatty acids (found in extra virgin oil, fish, and nuts), and bioactive compounds with antioxidant and anti-inflammatory properties (e.g., polyphenols, phytosterols, and carotenoids, found in fruit, vegetables, legumes, and cereals) [15,21,25,61]. In addition, adherence to the MD has been associated with enhanced GLP-1 activity and increased production of short-chain fatty acids, both of which are suggested to have a positive impact on satiety, insulin sensitivity, and pancreatic β-cell function [21,25].

A principal observation of this study is the marked inverse association between MD adherence and tissue-bound AGEs, as assessed via skin and lens autofluorescence. These associations remained significant after adjusting for clinically relevant covariates. Importantly, these associations were not materially altered after additionally adjusting for metformin and insulin use, indicating that they were not explained by differences in glucose-lowering treatment between groups. The findings concur with earlier cross-sectional reports of an inverse MD-adherence–SAF relationship in T2DM individuals in Croatia [32], healthy individuals aged 18–30 years [28], and middle-aged individuals without known cardiovascular disease or T2DM [27].

This relationship may be mediated through several biological mechanisms. The antiglycation effects of the MD may, in part, be attributed to its low content of dietary AGEs [15]. Evidence from dietary AGE databases suggests that the AGE intake can be substantially reduced by increasing the consumption of fruit, vegetables, whole grains, legumes, fish, and low-fat dairy products, while lowering the consumption of red meat, solid fats, and processed foods [62]. These dietary characteristics are all consistent with the MD [17,18,20].

The MD may also protect against glycation by reducing endogenous AGE formation. Its high content of dietary fiber and unrefined cereals may slow intestinal glucose absorption, thereby lowering both glucose levels and glycemic variability. Additionally, phenolic compounds present in foods typical of the MD act as electron donors, contributing to the reduction in free radicals and oxidative stress. The use of olive oil as the primary fat source is associated with a higher intake of monounsaturated fatty acids, which play an important role in attenuating inflammatory processes. In parallel, the MD has been associated with the upregulation of protective genes, such as receptor for advanced glycation end-products type 1 (*AGER1*) and glyoxalase I (*GloxI*), and the downregulation of genes involved in AGE-related damage, such as *RAGE*, thereby contributing to reduced endogenous AGE production [15,21,25].

By contrast, serum AGEs showed no significant association with MD adherence. Nevertheless, existing evidence supports an inverse relationship between MD adherence and circulating AGEs. Specifically, intervention trials have reported MD-induced reductions in circulating AGEs, both in healthy individuals and in individuals with CHD and T2DM [29,30,31].

One reason for the absent serum-AGE association may be that circulating AGEs are more susceptible to acute metabolic changes, such as those occurring in the postprandial state. Furthermore, in individuals with DM, impaired kidney function may limit the renal clearance of AGEs [13,45], although no association was observed between eGFR and serum AGEs in the present study.

In contrast, proteins in tissues, such as skin collagen, nerve myelin, and lens crystallins are characterized by slow turnover, making them more prone to AGE accumulation [40,44]. As a result, circulating AGE levels may not accurately reflect overall AGE accumulation in the body, supporting the use of tissue-bound AGEs as markers of long-term glycation [44,45].

Collectively, these observations suggest that the stronger associations observed with tissue AGEs may better reflect the long-term impact of dietary habits on glycation and oxidative burden.

The present findings have clinical implications, supporting the potential role of the MD in improving glycemic control and lowering glycation burden in individuals with T2DM. Given the involvement of AGEs in diabetes-related complications, the observed association between higher MD adherence and lower tissue AGE accumulation may indicate a broader beneficial effect of this dietary pattern on metabolic and vascular health. Hyperglycemia promotes oxidative stress, inflammation, and the production of AGEs, all of which can contribute to insulin resistance and endothelial dysfunction [63]. These findings support the MD as a potentially useful non-pharmacological approach within diabetes management. In addition, since AGE formation is strongly influenced by chronic hyperglycemia [9], it is plausible that the positive impact of the MD on AGEs is, at least partly, mediated through improved glycemic control.

A key strength is the study’s novelty: skin, ocular, and serum AGEs were simultaneously assessed in relation to MD adherence for the first time. Moreover, the study provides novel data on the relationship between MD adherence and AGE levels in individuals with T2DM in Greece. Furthermore, the current findings contribute to the existing literature on the determinants of MD adherence in this clinical population. The use of a validated dietary assessment score [47], along with simple and non-invasive techniques for the evaluation of AGEs [45] enhances the reliability and applicability of the results. Finally, the inclusion of multiple regression models allowed for the identification of independent associations between MD adherence and tissue-bound AGEs.

Several limitations warrant mention. The cross-sectional design rules out causal inference, and the modest sample size may have constrained statistical power. Relatedly, the treatment-adjusted models were based on modest sample sizes (*n* = 47 for skin AGEs and *n* = 32 for ocular AGEs); the limited statistical power, particularly for the smaller ocular subset, likely explains why the ocular association became borderline after additionally adjusting for metformin and insulin. Sample homogeneity and the lack of a control group may further constrain the interpretation of the findings and the identification of potential determinants. Reliance on a dietary assessment index may also introduce recall bias. Finally, although key variables were included in the regression models, residual confounding cannot be excluded. In particular, dietary AGE intake, which may influence AGE levels, was not directly assessed in this study.

Future studies with larger samples and longitudinal designs are needed to allow for causal inference regarding the relationship between MD adherence and AGEs. Further investigation is warranted to examine the factors that may hinder higher adherence to the MD in this clinical population, given its established benefits in disease management.

## 5. Conclusions

In conclusion, in this sample of individuals with T2DM in Greece, adherence to the MD was low to moderate. Higher adherence was associated with female sex and lower HbA1c levels. In addition, MD adherence was independently and inversely associated with tissue-bound AGEs, as assessed by SAF and LAF. No significant association was observed with circulating AGEs. These findings support the role of the MD as part of a comprehensive strategy for the prevention and management of diabetes-related complications, particularly through its association with improved glycemic control and lower tissue AGE accumulation. Further research is needed to confirm these findings and clarify potential causal relationships.

## Figures and Tables

**Table 1:** Baseline biochemical characteristics of the study population.

Parameter	Value	Reference Range
Fasting plasma glucose (mg/dL)	120.0 (105.0–147.0)	70–100
HbA1c (%)	6.95 (6.2–7.5)	4.8–6.0
Total cholesterol (mg/dL)	137.5 (110.0–156.3)	140–200
LDL cholesterol (mg/dL)	60.0 (45.9–85.3)	<100
HDL cholesterol (mg/dL)	46.6 (39.9–54.2)	<40.0 (low)/>60.0 (high)
Triglycerides (mg/dL)	111.0 (75.8–166.5)	50–150
eGFR (mL/min/1.73 m^2^)	92.0 (67.5–99.8)	≥90.0
ACR (mg/g)	18.0 (5.1–77.8)	<30.0
25-hydroxy-vitamin D (ng/mL)	20.3 (15.1–28.2)	>30 (sufficiency)
Ferritin (ng/mL)	56.5 (24.1–123.0)	15–150
Vitamin B12 (pg/mL)	363.0 (295.0–443.0)	223–925
Folate (ng/mL)	7.2 (4.5–8.9)	3.98–26.8

Continuous variables are presented as median (interquartile range). Reference ranges correspond to those of the clinical laboratory of the hospital. ACR: Albumin-to-creatinine ratio; eGFR: estimated glomerular filtration rate; HbA1c: glycated hemoglobin; HDL: high-density lipoprotein; LDL: low-density lipoprotein.

**Table 2:** Characteristics of participants according to adherence to the Mediterranean diet.

Variable	MedDietScore ≤ 29.0 (*n* = 34)	MedDietScore > 29.0 (*n* = 27)	*p*-Value
Female sex, *n* (%)	10 (29.4)	16 (59.3)	**0.019** *
Age (years)	65.0 ± 10.3	66.0 ± 7.3	0.672 ^†^
Diabetes duration (years)	14.8 ± 9.16	15.4 ± 8.63	0.804 ^†^
Glucose-lowering medication, *n* (%)			
Metformin	24 (70.6)	26 (96.3)	**0.024** *
Insulin	19 (55.9)	7 (25.9)	**0.019** *
SGLT-2 inhibitors	20 (58.8)	14 (51.9)	0.586 *
DPP-4 inhibitors	5 (14.7)	7 (25.9)	0.274 *
GLP-1 RA	21 (61.8)	14 (51.9)	0.437 *
Current smoking status, yes, *n* (%)	10 (29.4)	6 (22.2)	0.582 *
BMI (kg/m^2^)	28.5 (25.4–30.1)	32.2 (28.2–35.3)	**0.012** ^#^
Waist circumference (cm)	104.0 (97.0–109.0)	111.0 (104.0–113.0)	**0.023** ^#^
Hip circumference (cm)	102.3 ± 9.32	104.8 ± 6.15	0.327 ^†^
WHR	1.02 (0.94–1.05)	1.06 (0.98–1.09)	0.147 ^#^
WHtR	0.61 ± 0.07	0.66 ± 0.07	**0.034** ^†^
HbA1c (%)	7.3 (6.45–8.45)	6.5 (5.9–7.1)	**0.002** ^#^
eGFR (mL/min/1.73 m^2^)	91.0 (64.5–100.5)	93.0 (77.2–99.0)	0.694 ^#^
Ocular AGEs (AU)	0.26 ± 0.06	0.22 ± 0.05	0.071 ^†^
Skin AGEs (AU)	3.0 (2.7–4.0)	2.8 (2.4–3.3)	0.131 ^#^
Serum AGEs (ng/mL)	37.7 (24.7–80.7)	27.6 (20.8–49.4)	0.107 ^#^

Categorical variables are presented as absolute (*n*) and relative (%) frequencies. Continuous variables are presented as mean ± standard deviation (SD) or median (interquartile range), as appropriate. *p*-values were calculated using Pearson’s chi-square test for categorical values and Student’s *t*-test or Mann–Whitney *U* test for continuous variables. Values shown in bold are statistically significant (*p* < 0.05). * Pearson’s chi-square test. ^†^ Student’s *t*-test. ^#^ Mann–Whitney *U* test. Reference values: HbA1c: 4.8–6.0%, eGFR ≥ 90 mL/min/1.73 m^2^. No established reference values exist for ocular, skin, and serum AGEs. AGEs: Advanced glycation end-products; AU: arbitrary units; BMI: body mass index; DPP-4: dipeptidyl-peptidase 4; eGFR: estimated glomerular filtration rate; GLP-1 RA: glucagon-like peptide-1 receptor agonist; HbA1c: glycated hemoglobin; SGLT-2: sodium–glucose cotransporter-2; WHR: waist-to-hip ratio; WHtR: waist-to-height ratio.

**Table 3:** Correlations of MedDietScore with demographic, anthropometric, clinical, and biochemical characteristics.

Parameter	*ρ* (Spearman)	*p*-Value
Sex (0 = male; 1 = female)	0.248	0.054
Age (years)	0.085	0.516
Diabetes duration (years)	0.035	0.793
Current smoking status (0 = no; 1 = yes)	−0.087	0.510
BMI (kg/m^2^)	0.185	0.156
Waist circumference (cm)	0.136	0.366
WHR	0.154	0.319
WHtR	0.183	0.225
HbA1c (%)	−0.437	**<0.001**
eGFR (mL/min/1.73 m^2^)	0.047	0.722
Ocular AGEs (AU)	−0.435	**0.013**
Skin AGEs (AU)	−0.309	**0.033**
Serum AGEs (ng/mL)	−0.255	0.060

*ρ* = Spearman’s rank correlation coefficient. Values shown in bold are statistically significant (*p* < 0.05). AGEs: Advanced glycation end-products; AU: arbitrary units; BMI: body mass index; eGFR: estimated glomerular filtration rate; HbA1c: glycated hemoglobin; WHR: waist-to-hip ratio; WHtR: waist-to-height ratio.

## Data Availability

The original contributions presented in this study are included in the article. Further inquiries can be directed to the corresponding authors.

## Abbreviations

The following abbreviations are used in this manuscript:

ABI	Ankle–brachial index
ACR	Albumin-to-creatinine ratio
AGER1	Receptor for advanced glycation end-products type 1
AGEs	Advanced glycation end-products
AU	Arbitrary unit
BMI	Body mass index
CHD	Coronary heart disease
CKD-EPI	Chronic Kidney Disease Epidemiology Collaboration
DM	Diabetes mellitus
DPP-4	Dipeptidyl-peptidase-4
eGFR	Estimated glomerular filtration rate
ELISA	Enzyme-linked immunosorbent assay
Glox1	Glyoxalase 1
GLP-1 RA	Glucagon-like peptide 1 receptor agonist
HbA1c	Glycated hemoglobin
HDL	High-density lipoprotein
IDF	International Diabetes Federation
LAF	Lens autofluorescence
LDL	Low-density lipoprotein
MD	Mediterranean diet
MedDietScore	Mediterranean Diet Score
RAGE	Receptor for advanced glycation end-products
SAF	Skin autofluorescence
SD	Standard deviation
SGLT-2	Sodium–glucose cotransporter-2
SPSS	Statistical Package for the Social Sciences
T2DM	Type 2 diabetes mellitus
VIF	Variance inflation factor
WHR	Waist-to-hip ratio
WHtR	Waist-to-height ratio
